# A dataset of four probiotic *Bifidobacterium* strains genome assemblies

**DOI:** 10.1016/j.dib.2020.106710

**Published:** 2020-12-31

**Authors:** Aleksei A Korzhenkov, Alina V Tepliuk, Konstantin V Sidoruk, Konstantin E Voyushin, Maksim V Patrushev, Ilya V Kublanov, Stepan V Toshchakov

**Affiliations:** a National Research Center “Kurchatov Institute”, Moscow, Russia; b Federal Institution “State Research Institute of Genetics and Selection of Industrial Microorganisms” of the National Research Center “Kurchatov Institute”, Moscow, Russia; c Winogradsky Institute of Microbiology, Research Center of Biotechnology of the Russian Academy of Sciences, Moscow, Russia; d Albiogen, Moscow, Russia

**Keywords:** Microbial genomics, Probiotics, Bacteriocins, Bifidobacteria, Human milk oligosaccharides

## Abstract

A dataset of four draft genome sequences of *Bifidobacterium* strains is presented. All four genome assemblies are high-quality drafts characterized by high completeness and low contamination levels. GC content of the genomes varied in the range between 59.27% and 62.77%. Genome sequences were annotated for further functional and taxonomical analyses of the respective *Bifidobacterium* strains. Genetic determinants of probiotic capabilities, including the genes, related to utilization of human milk oligosaccharides and mucin, as well as the genes, encoding bile salt hydrolase were identified. The genome of *B. bifidum* VKPM=Ac-1784 has been shown to possess two bacteriocin gene clusters. The dataset expands knowledge on genomic diversity of probiotic strains of *Bifidobacterium* genus. The dataset is available under PRJNA656137 accession number in NCBI database and under zyv26t6x5r accession number in Mendeley Data repository.

## Specifications Table

**Table:** 

Subject	‘Microbiology’
Specific subject area	Microbial genomics of beneficial bacteria
Type of data	Genome assemblies
How data were acquired	Genomic DNA was extracted, NGS libraries were prepared and sequenced. Raw reads were processed and assembled into contigs. Genome assemblies were annotated.
Data format	FASTA format for genome sequences
	GenBank format for genome annotations
Parameters for data collection	DNA extraction was performed by bead-beating following standard phenol-chloroform method. Fragment NGS libraries were prepared using NEBNext® Ultra™ II DNA Library Prep Kit for Illumina®, according to manufacturer's instructions . Reads processing, genome assembly and annotation were performed with default settings of publicly available ZGA pipeline (https://github.com/laxeye/zga).
Description of data collection	Genomic DNA was isolated from the pure cultures of described *Bifidobacterium* strains. Four fragment genomic libraries (one for each strain) were prepared. Raw data were acquired by Illumina MiSeq system using 2 × 250 bp paired-end sequencing chemistry
	Raw sequencing reads were subjected to quality control, quality trimming, adapter trimming and filtering, overlapped read pairs were merged. De novo assembly of draft genome sequences was performed using SPAdes ver. 3.12. Genome assembly quality was assessed using CheckM ver. 1.1.2. Genome annotation was performed using DFAST ver. 1.2.6. Average nucleotide identity between genome assemblies was assessed using ani.rb script.
Data source location	The following microorganisms were the source of the genomic DNA: *B. adolescentis* VKPM=Ac-1245, *B. bifidum* VKPM=Ac-1579, *B. longum* subsp. longum VKPM=Ac-1635 and *B. bifidum* VKPM=Ac-1784. The strains are deposited in the Russian National Collection of Industrial Microorganisms (VKPM), Moscow, Russian Federation.
Data accessibility	Repository name: NCBI WGS
	Data identification number: JACTOC000000000, JACTOD000000000, JACTOE000000000, JACTOF000000000
	Direct URL to data:
	https://www.ncbi.nlm.nih.gov/Traces/wgs/JACTOC01
	https://www.ncbi.nlm.nih.gov/Traces/wgs/JACTOD01
	https://www.ncbi.nlm.nih.gov/Traces/wgs/JACTOE01
	https://www.ncbi.nlm.nih.gov/Traces/wgs/JACTOF01
	Repository name: Mendeley Data
	Data identification number: zyv26t6x5r
	Direct URL to data: https://data.mendeley.com/datasets/zyv26t6x5r

## Value of the Data

• The dataset provides information on genomic diversity of Bifidobacterium genus useful for phylogenetic analysis of Bifidobacterium strains and genome-inspired development of new probiotic formulations.
• The data may broaden current knowledge on biology of microorganisms, regarded as probiotics, their metabolism, ecology and interactions, as well as their outcome on the human health. The data is beneficial for scientist in the fields of microbiology, nutrition, biotechnology, molecular biology.
• The dataset contains information on genes, responsible for mucin and human milk oligosaccharides utilization which may be of interest for biomedicine, nutritional and food science, as well as data on putative bacteriocin synthesis clusters are of interest for new approaches to the treatment of infection diseases.

## 1 Data Description

Representatives of *Bifidobacterium* genus, firstly discovered more than a century ago in the feces of the breast-fed infants [1], arouse significant research interest during last decades, due to their pronounced probiotic properties. Since the beginning of genomic era in early 2000s several extensive studies of genomic determinants of probiotic features were published [2], [3], [4] and implementation of genome-based phylogeny allowed thorough evolutionary reconstruction of *Bifidobacterium* genus [5]. Nevertheless, high level of genome mobility in gut microbiota results in increased genomic and functional diversity of *Bifidobacterium* strains. This emphasizes the importance of genomic studies of new *Bifidobacterium* strains, possessing genetic determinants of probiotic-related traits. In this report a dataset of four probiotic *Bifidobacterium* strains genome assemblies is presented. All assembled genomes have high completeness, low contamination and low to moderate number of contigs (Table 1). That gives an opportunity to perform robust and reliable functional analysis, including identification of probiotic-related genomic loci.

**Table 1:** Results of genome sequencing, assembling and gene prediction.

Strain	*B. adolescentis* VKPM=Ac-1245	*B. bifidum* VKPM=Ac-1579	*B. longum subsp. longum* VKPM=Ac-1635	*B. bifidum* VKPM=Ac-1784
NGS raw read pairs	1760060	1458774	248380	1708375
Assembly length, bp	2218534	2231702	2240429	2267064
Assembly N50, bp	1127661	348994	209044	517354
GC content, %	59.27	62.77	60.07	62.44
Contig count	12	41	19	15
CDS	1887	1858	1827	1883
tRNA	62	53	53	53
Genome completeness, %	100	99.9	100	99.9
Genome contamination, %	0	0	0.25	0

**Fig. 1:**
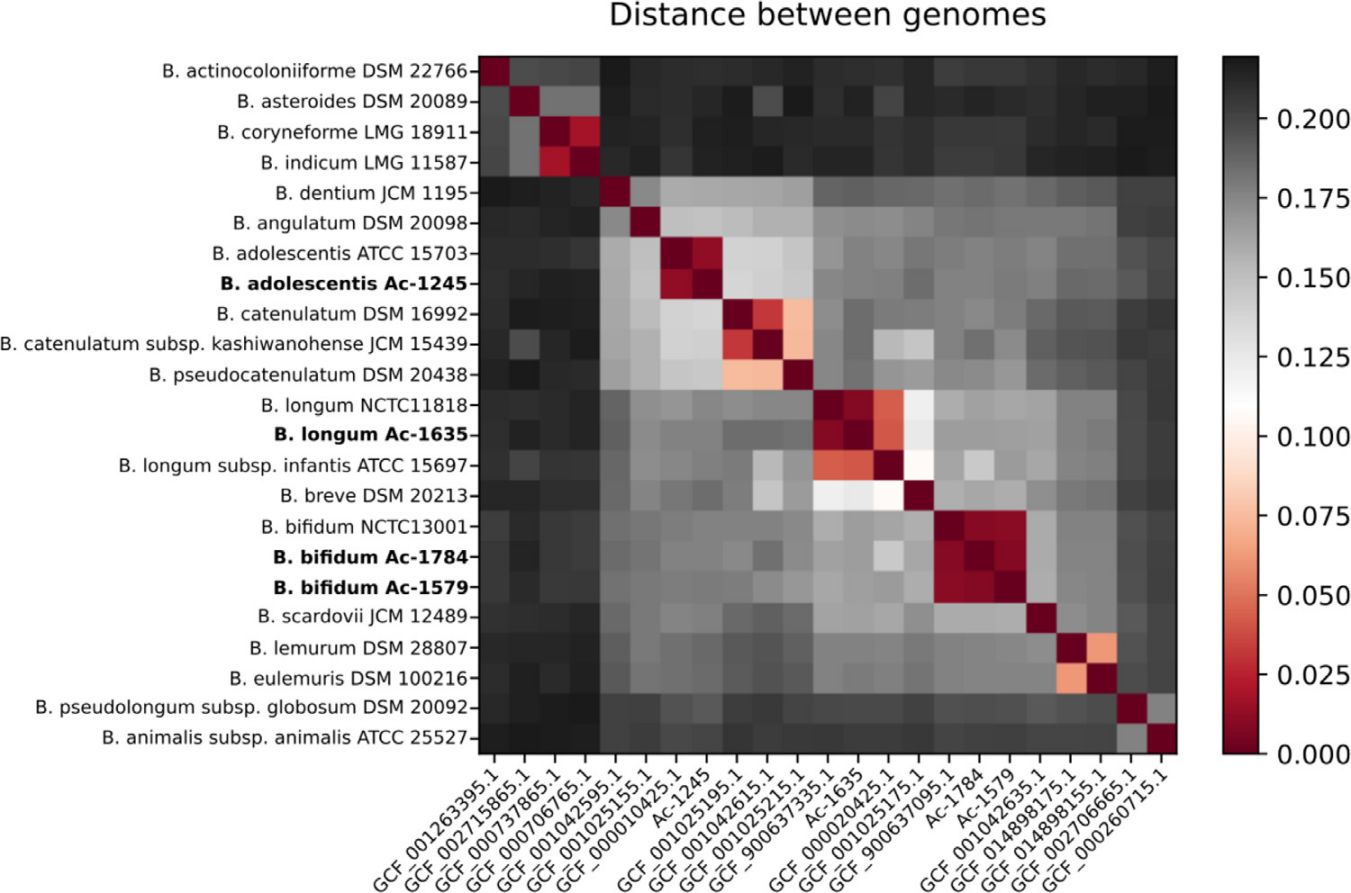
Heatmap illustrating Intergenomic distance between representative strains of *Bifidobacterium* species and the strains sequenced in present study.

**Fig. 2:**
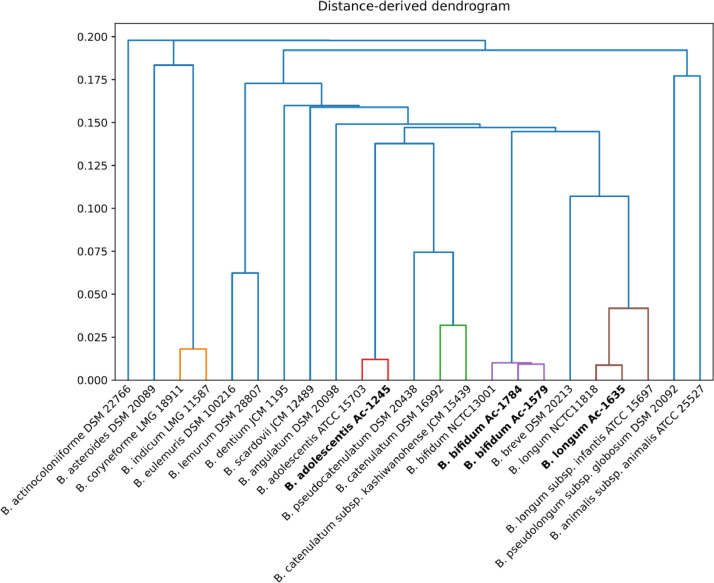
Dendrogram constructed on intergenomic distance between representative strains of *Bifidobacterium* species and the strains sequenced in present study.

Analysis of nucleotide identity-based intergenomic distance between studied strains and complete representative genomes of *Bifidobacterium* genus allowed to perform exact taxonomic assignment (Fig. 1, Fig. 2, Supplementary table S1). Search for novel genes and gene clusters, presented in studied strains, performed by pangenome analysis, revealed 24 unique genes in Ac-1245 and 7 unique genes in Ac-1579 genomes (Supplementary table S2). Interestingly, cluster of unique CDSs in Ac-1245 genome, which was likely acquired from representatives of class *Coriobacteriia* (GPVLNQ_01920-GPVLNQ_01960, Supplementary Table S2), possess a component of PhoP–PhoQ signal transduction system, known to be involved in response to low Mg^2+^ concentration or mildly acidic pH in several bacterial pathogens [6].

Search of antibiotic-related genes using antiSMASH revealed two bacteriocin gene clusters encoding for antibiotic synthetase, ABC transporter and antibiotic peptide in Ac-1784 genome (Supplementary table S3). All sequenced genomes have a copy of choloylglycine hydrolase (bile salt hydrolase) which may affect metabolism of the host and alter the ratio between its fat and carbohydrate metabolism [7]. Search of genes related to utilization of host-produced hydrocarbons (HPHC) [8] and human milk oligosaccharides (HMO) [2] revealed different gene repertoire in the studied strains: only Ac-1579 and Ac-1784 genomes possess genes, which may be responsible for HMO and HPHC utilization (Supplementary Table S4).

## 2 Experimental Design, Materials and Methods

### 2.1 Strain cultivation

*Bifidobacterium* strains studied in this work were originally isolated from human feces and cultivated on Blaurock (Ac-1579) or Bifidum media (Ac-1245, Ac-1635 and Ac-1784).

### 2.2 DNA extraction, library preparation and sequencing

Genomic DNA was isolated from *Bifidobacterium* strains previously deposited in Russian National Collection of Industrial Microorganisms (VKPM, https://vkpm.genetika.ru/). Cell lysis was performed mechanically by bead-beating with glass beads on the Disruptor Genie™ (Scientific Industries, USA) cell disruptor. DNA was extracted using phenol-chloroform method [9]. DNA quality and integrity were assessed with agarose gel electrophoresis and by measurement of ratios of A260/A280 and A260/A230 ratios with Nanodrop 1000 spectrophotometer (Thermo Fisher Scientific, USA).

DNA was fragmented using Covaris S2 ultrasonication device (Covaris Inc., USA) to achieve 500 bp mean fragment length. DNA libraries were prepared using NEBNext® Ultra™ II DNA Library Prep Kit for Illumina® (New England BioLabs, USA) according to manufacturer's instructions. Paired-end de novo genome sequencing was performed on MiSeq® System (Illumina, USA) using MiSeq Reagent kit v2 (Illumina, USA).

### 2.3 Genome assembly and annotation

Genome assembly and annotation were performed using ZGA pipeline (https://github.com/laxeye/zga/): low quality bases and adapter sequences were trimmed out from reads, short reads were filtered out with BBduk [10], overlapping paired reads were merged using BBmerge [10], genomes were assembled using SPAdes with k-mer based error correction [11]. Genome completeness were assessed using CheckM [12], genome annotation was performed using DFAST [13], genome assembly metrics were determined using QUAST [14]. Search of putative antibiotics-related genes was conducted with antiSMASH web server [15] and Bagel4 web-server [16]. Sequences of genes involved in HPHC [8] and HMO [2] utilization were acquired from genome assemblies from above mentioned studies. Pairwise alignment of protein sequences was conducted with BLASTp [17], hits with e-value less than 1e-6, identity less than 50% or alignment coverage less than 50% were discarded.

Unique genes were identified using next workflow. All available on 10/1/2020 genome assemblies of *Bifidobacterium* were downloaded from NCBI GenBank. For all downloaded genomes ANI values against newly sequenced strains were calculated using FastANI [18]. For each species (*B. adolescentis* (Ac-1245), *B. bifidum* (Ac-1579, Ac-1784), *B. longum* (Ac-1635)) downloaded genomes were selected basing on ANI > = 95%. Genomes with ANI less than 95% to any of newly sequenced genomes were discarded, resulting genomic datasets had size of 318, 111 and 752 genome sequences for *B. adolescentis, B. bifidum* and *B. longum* respectively. For each group of genomes protein coding sequences were predicted using prodigal [19] and orthologous genes were detected using proteinortho [20]. Singletons from newly sequenced genomes were aligned against NCBI database using web-based BLASTp [17] against nr and viral nr databases.

Taxonomical placement of newly sequenced strains was verified using calculation of average nucleotide identity (ANI) between genomic sequences of studied bacteria and complete genomes of type strains of genus *Bifidobacterium*, available in NCBI GenBank using ani.rb script (https://github.com/lmrodriguezr/enveomics), genomic distance was calculated as 1 minus ANI and visualized using Python script (https://github.com/laxeye/genomic-utilities/blob/master/genomic_distance_viz.py).

## Declaration of Competing Interest

The authors declare that they have no known competing financial interests or personal relationships which have, or could be perceived to have, influenced the work reported in this article.

## Appendix Supplementary materials

Image, application 1

Image, application 2

Image, application 3

Image, application 4

## Footnotes

Supplementary material associated with this article can be found in the online version at doi:10.1016/j.dib.2020.106710.
